# ^1^H/^13^C chemical shifts and cation binding dataset of the corticosteroid Prednisolone titrated with metal cations

**DOI:** 10.1016/j.dib.2019.104620

**Published:** 2019-10-09

**Authors:** Kathleen Joyce D. Carillo, Danni Wu, Su-Ching Lin, Shen-Long Tsai, Jiun-Jie Shie, Der-Lii M. Tzou

**Affiliations:** a Taiwan International Graduate Program - SCST, Academia Sinica, Nankang, Taipei 11529, Taiwan, ROC; b Department of Applied Chemistry, National Chiao-Tung University, Hsinchu 30013, Taiwan, ROC; c Institute of Chemistry, Academia Sinica, Nankang, Taipei 11529, Taiwan, ROC; d Chemical Engineering Department of NTUST, Taipei 10607, Taiwan, ROC; e Department of Applied Chemistry, National Chia-Yi University, Chia-Yi 60004, Taiwan, ROC

**Keywords:** Prednisolone, Nuclear magnetic resonance, Cation binding, Binding affinity

## Abstract

We here reported the ^1^H/^13^C chemical shifts, binding affinity and binding free energy of 1,4-pregnadiene-11β,17α,21-triol-3,20-dione (Prednisolone; Prd) interacting with metal cations. Six different Prd/Ni or Co mixtures were examined at different molar ratios (1:0, 1:0.1, 1:0.2, 1:0.3, 1:0.4 and 1:0.5). In this analysis, the ^1^H and ^13^C chemical shifts were measured for the Prd/cation mixtures using a Bruker AV 500 MHz spectrometer (Bruker BioSpin GmbH, Rheinstetten, Germany), equipped with a 5 mm z-gradient Prodigy BBO 500 MHz probehead at 298 K, and simulation of the ^1^H spectra were determined from the Daisy software package (Bruker BioSpin GmbH). Binding affinity and free energy values were deduced from the ^13^C NMR peak intensities involved in the cation interaction, for more insight on the steroid/cation interactions please see Magnesium and Calcium Reveal Different Chelating Effects in a Steroid Compound: A Model Study of Prednisolone Using NMR Spectroscopy [1].

**Table:** Specifications Table

Subject area	Chemistry, Biochemistry
More specific subject area	Steroids, Cortisone, Steroidal drugs
Type of data	Table, NMR spectra
How data was acquired	Bruker AV 500 MHz spectrometer (Bruker BioSpin GmbH, Rheinstetten, Germany)
Data format	Analyzed and raw data
Experimental factors	Prednisolone incubated with metal cation at different molar ratios
Experimental features	Binding affinity constant and binding energy were determined using ^13^C peak intensity of Prednisolone measured in the presence of cations
Data source location	Taipei, Taiwan
Data accessibility	Analyzed: Within the Data in Brief article Raw Data Repository name: Mendeley Data Data identification number: https://doi.org/10.17632/g3nf3k426t.1 Direct URL to data: https://data.mendeley.com/datasets/g3nf3k426t/1
Related research article	Carillo, K. D., Wu, D., Lin, S. C., Tsai, S. L., Shie, J. J., & Tzou, D. L. M. (2019). Magnesium and Calcium Reveal Different Chelating Effects in a Steroid Compound: A Model Study of Prednisolone Using NMR Spectroscopy. Steroids, 108429. https://doi.org/10.1016/j.steroids.2019.108429

**Table:** 

**Value of the data** • The cation binding data are useful for corticosteroid drug metabolism and pharmacokinetic analysis • Chemical biologists and medicinal chemists could benefit from the cation/Prednisolone chelation dataset • Our data can be used for assessing adverse effects upon treatment of Prednisolone as well as other corticosteroid related drugs in animal models • Cation binding datasets are physiologically valuable for corticosteroid drug developments to avoid cation interaction

## 1 Data

^1^H and ^13^C NMR measurements of corticosteroid Prednisolone (Prd) titrated with four metal cations, including Co^2+^, Ni^2+^, Mg^2+^ and Ca^2+^, are reported (Table 1, Table 2). ^1^H spectral pattern simulations [1] were carried out to identify each of these ^1^H chemical shifts (Supplementary Fig. S5). The metal cation induced shifting effect was analyzed by the ^1^H and ^13^C chemical shift deviations, reported in Table 3. Metal cation binding affinity and binding free energy deduced from binding equilibrium analysis corresponding to the two metal cation binding sites are listed in Table 4.

**Table 1:** ^1^H chemical shift assignments of Prd in the presence of metal cations.^a^^,^^b^

Proton	^1^H chemical shifts (ppm)			
	Co^2+^	Ni^2+^	Mg^2+^	Ca^2+^
1	7.433	7.482	7.461	7.484
2	6.180	6.256	6.230	6.254
4	5.932	6.014	5.986	6.011
6α (ax)	2.318	2.383	2.354	2.374
6β (eq)	2.555	2.660	2.631	2.649
7α (eq)	1.075	1.138	1.107	1.124
7β (eq)	2.089	2.163	2.141	2.162
8β (ax)	2.115	2.185	2.161	2.173
9α (ax)	0.963	1.022	0.991	1.006
11β (eq)	4.358	4.413	4.388	4.407
12α (ax)	1.573	1.609	1.589	1.605
12β (eq)	1.958	2.006	1.972	1.998
14α (ax)	1.665	1.725	1.701	1.731
15α (ax)	1.711	1.792	1.756	1.783
15β (eq)	1.353	1.440	1.408	1.440
16α (eq)	1.393	1.475	1.438	1.528
16β (ax)	2.601	2.728	2.690	2.716
Me-18	1.439	1.500	1.472	1.484
Me-19	0.825	0.923	0.888	0.933
21a	4.393	4.266	4.240	4.376
21b	4.732	4.631	4.605	4.772

a The sample was dissolved in CD_3_OD. ^1^H chemical shifts are in units of ppm referenced to the *d*4-methanol resonance at 4.87 ppm, within an uncertainty of ±0.001 ppm.

b Solution ^1^H NMR chemical shift assignments of Prd in the presence of CoCl_2_(5 mM), or NiCl_2_(5 mM), MgCl_2_(84 mM) or CaCl_2_(84 mM) determined from HSQC and COSY experiments. The assignments of Prd/Mg^2+^and Prd/Ca^2+^ reported previously are listed for comparison [1].

**Table 2:** ^13^C chemical shift assignments of prednisolone in the presence of metal cations.^a^

Carbon	^13^C chemical shifts (ppm)^b^			
	Co^2+^	Ni^2+^	Mg^2+^	Ca^2+^
C1	160.23	160.32	160.35	160.44
C2	128.00	128.17	127.92	127.96
C3	189.78	189.48	189.14	189.17
C4	122.86	122.82	122.59	122.64
C5	174.90	174.99	175.03	175.14
C6	33.34	33.45	33.34	33.34
C7	35.69	35.83	35.73	35.72
C8	32.79	32.93	32.82	32.84
C9	57.43	57.58	57.48	57.45
C10	46.16	46.29	46.22	46.24
C11	70.86	70.99	70.88	70.83
C12	40.70	40.82	40.70	40.61
C13	48.68	NA^c^	NA^c^	NA^c^
C14	52.94	53.06	52.94	53.00
C15	24.96	25.01	25.01	25.02
C16	34.61	34.76	34.67	34.87
C17	90.40	90.53	90.42	90.36
C18	17.79	17.94	17.85	17.91
C19	21.65	21.80	21.70	21.69
C20	214.40	213.10	213.14	216.19
C21	67.42	67.89	67.78	68.58

a The sample was dissolved in CD_3_OD. ^13^C chemical shifts are in units of ppm referenced to the *d*4-methanol resonance (methyl) at 49.15 ppm, within an uncertainty of ±0.01 ppm.

b The^13^C chemical shifts of Prd were assigned in the presence of CoCl_2_(5 mM), or NiCl_2_(5 mM), MgCl_2_(84 mM) or CaCl_2_(84 mM), respectively. The assignments of Prd/Mg^2+^and Prd/Ca^2+^ reported previously are listed for comparison [1].

c NA: Due to signal overlapping with solvent peak, the^13^C chemical shift is not available.

**Table 3:** Characterization of cation chelation induces shifting effects of Prd in the presence of metal cations.^a^

Metal cation induced shifting effect (M^−1^)									
Atom	Co^2+^	Ni^2+^	Mg^2+^	Ca^2+^	Atom	Co^2+^	Ni^2+^	Mg^2+^	Ca^2+^
H2	−13.83	−3.93	−0.56	−0.18	C2	250.	217.	−0.55	−0.51
H4	−14.40	−4.01	−0.58	−0.19	C4	263.	227.	−0.49	−0.14
H15α	−13.78	−4.35	−0.60	−0.25	–	–	–	–	–
H15β	−13.15	−4.64	−0.60	−0.22	–	–	–	–	–
H16α	−13.14	−5.23	−0.57	0.30	C16	−28.	−8.	−0.41	1.50
H16β	−17.02	−4.04	−0.60	−0.33	–	–	–	–	–
–	–	–	–	–	C17	86.	−121.	−0.36	−0.77
–	–	–	–	–	C20	89.	−112.	1.45	36.7
H21	14.25	−3.01	−0.51	2.05/0.48	C21	−69.	−105.	0.30	7.50

a The metal cation induced shifting effect of Prd/Mg^2+^and Prd/Ca^2+^reported previously are listed for comparison [1], for Prd/Mg^2+^ and Prd/Ca^2+^ spectra see Supplementary Figs. S1–S4.

**Table 4:** Cation binding affinity and binding free energy deduced from ^13^C NMR signals of Prd/metal cation complexes.^a^

Atom	Binding affinity			Binding free energy (kJ/mol)		
	Co^2+^( × 10^2^ M^−1^)	Ni^2+^( × 10^2^ M^−1^)	Mg^2+^(M^−1^)^b^	Co^2+^	Ni^2+^	Mg^2+^^b^
C2 (C3)	40.6 ± 2.0	18.0 ± 0.6	3.9 ± 0.1	−20.6 ± 1.0	−18.6 ± 0.6	−1.7 ± 0.1
C4 (C5)	40.9 ± 2.7	15.0 ± 1.0	13.3 ± 2.3	−20.6 ± 1.4	−18.1 ± 1.2	−6.4 ± 1.1
C17	113.0 ± 6.6	35.8 ± 4.4	3.8 ± 0.4	−23.1 ± 1.5	−20.3 ± 0.3	−3.3 ± 0.1
C20	135.0 ± 7.3	79.4 ± 4.6	19.1 ± 0.1	−23.6 ± 1.3	−22.3 ± 1.3	−7.3 ± 0.3
C21	45.0 ± 2.4	75.3 ± 3.0	14.7 ± 0.2	−20.8 ± 1.1	−22.1 ± 0.9	−3.6 ± 0.2

a The binding affinities were deduced from the curve fitting analysis of the Prd/Co^2+^ and Prd/Ni^2+^complexes [3]. The binding free energies were calculated from binding affinity using the free energy equation ΔG = -RTlnK_d_, in which R is gas constant, T absolute temperature and K_d_ is the binding affinity.

b The binding affinity or binding free energy deduced from C3, C5, C17, C20 and C21 signals of Prd/Mg^2+^ mixtures reported previously are listed for comparison [1].

## 2 Experimental design, materials and methods

The 1,4-Pregnadiene-11β, 17α,21-triol-3,20-dione (Prd) (purity ≥ 99%), CoCl_2_, NiCl_2_, MgCl_2_ and CaCl_2_ reagents were all purchased from Sigma-Aldrich (St, Louis, MO, USA) and used without further purification. Before the Prd/Co, Prd/Ni, Prd/Mg and Prd/Ca mixtures were prepared, both the steroid and metal salts were dried by lyophilizing for 3 hours prior to weighting, in order to remove all adsorbed moisture [2]. After lyophilization, a series of Prd/metal salt (CoCl_2_ or NiCl_2_) mixtures with 1:0, 1:0.1, 1:0.2, 1:0.3 and 1:0.4 and 1:0.5 molar ratios were then prepared. In the case of Prd/Mg^2+^ and Prd/Ca^2+^, higher molar ratios were used, namely 1:2.5, 1:5, 1:7.5 and 1:10. Around 3 mg of Prd was weighted and mixed with increasing amount of CoCl_2_, NiCl_2_, MgCl_2_ or CaCl_2_ dissolved in 500 μL of anhydrous *d*_4_-methanol (Sigma-Aldrich).

For all samples, the 1D ^1^H and ^13^C NMR experiments were performed using a Bruker AV 500 MHz spectrometer (Bruker BioSpin GmbH, Rheinstetten, Germany), equipped with a 5 mm z-gradient Prodigy BBO 500 MHz probehead operating at 298 K. All the spectra acquired were then processed using the TopSpin 3.5 software (Bruker BioSpin GmbH). In order to determine the ^1^H chemical shifts, analysis was done using the Daisy software package (Bruker BioSpin GmbH) available in the TopSpin3.5 software.

The metal cation shifting effects were deduced from the plots of ^1^H or ^13^C chemical shift deviations against cation concentration for Prd/metal cation mixtures. And the metal cation binding affinity and binding free energy were determined from the curve fitting of the ^13^C signal intensity variation against cation concentration using multiple binding equilibrium simulation [3].
