# Supplementary material for: 1H/13C chemical shifts and cation binding dataset of the corticosteroid Prednisolone titrated with metal cations
Source: Data Brief. 2019 Oct 9;27:104620. doi: 10.1016/j.dib.2019.104620 (PMC6820118; doi:10.1016/j.dib.2019.104620)
Supplement: Multimedia component 1 [file mmc1.doc]

# ^1^H/^13^C chemical shifts and cation binding dataset of the corticosteroid Prednisolone titrated with metal cations

# Kathleen Joyce D. Carillo,^†,#,§,‡^ Danni Wu,^§,‡^ Su-Ching Lin, ^§^ Shen-Long Tsai,^&^ Jiun-Jie Shie,^§^ and Der-Lii M. Tzou ^§, ║^,*

# ^†^Taiwan International Graduate Program - SCST, Academia Sinica, Nankang, Taipei 11529, Taiwan ROC

# ^#^The Department of Applied Chemistry, National Chiao-Tung University, Hsinchu 30013, Taiwan ROC

# ^§^Institute of Chemistry, Academia Sinica, Nankang, Taipei 11529, Taiwan ROC

# ^&^Chemical Engineering Department of NTUST, Taipei 10607, Taiwan ROC

*^║^*Department of Applied Chemistry, National Chia-Yi University, Chia-Yi 60004, Taiwan, ROC

^‡^ These authors contributed equally.

**Supplementary information**

# *To whom correspondence should be addressed: Dr. Der-Lii M. Tzou, Institute of Chemistry, Academia Sinica 128, Academia Rd., Sec. 2, Nankang, Taipei 11529, Taiwan, Republic of China, Tel: +886 2 5572 8524, fax: +886 2 5572 1237, E-mail: [**tzougate@gate.sinica.edu.tw**](mailto:tzougate@gate.sinica.edu.tw)

#
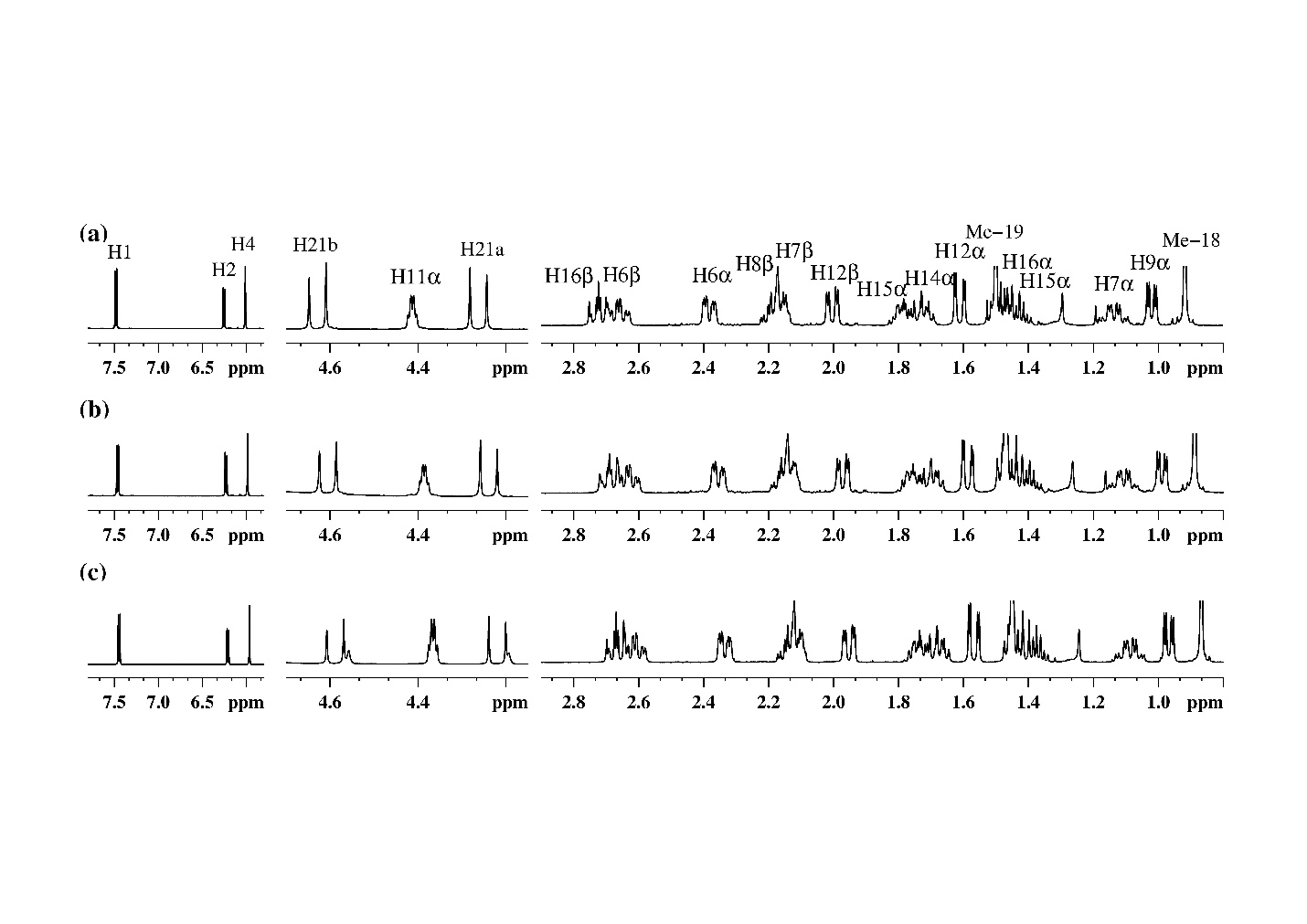


**Figure S1.** Solution ^1^H NMR spectra of Prd/Mg^2+^ mixtures at three different Prd/Mg^2+^ molar ratios: (a) 1:2.5, (b) 1:5.0, (c) 1:7.5. Systematic upfield shifts occurred for all ^1^H resonances as a function of Mg^2+^ concentration. The ^1^H chemical shift assignments of the Prd/Mg^2+^ mixtures are indicated at the top. All spectra are presented in three different ranges: δ=0.80-2.80, 4.10-4.70, and 5.90-7.60 ppm.

**
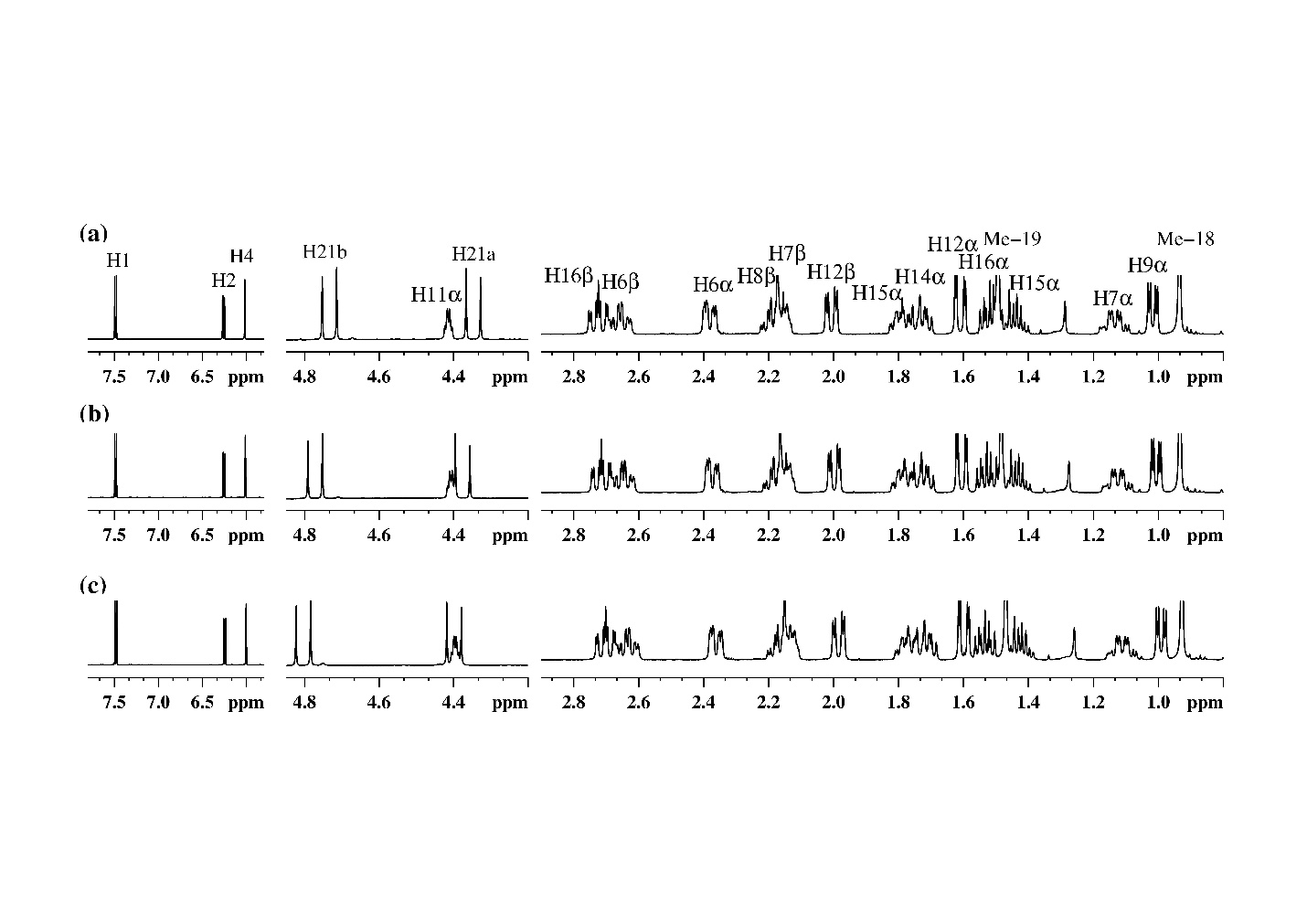
**

**Figure S2.** Solution ^1^H NMR spectra of Prd/Ca^2+^ mixtures at three different Prd/Ca^2+^ molar ratios: (a) 1:2.5, (b) 1:5.0, (c) 1:7.5. Systematic downfield shifts occurred for H21b, H21a and H16α and upfield shifts for the rest of the ^1^H resonances were observed as a function of Ca^2+^ concentration. The ^1^H chemical shift assignments of the Prd/Ca^2+^ mixtures are indicated at the top. All spectra are presented in three different ranges: δ=0.80-2.80, 4.10-4.70, and 5.90-7.60 ppm.


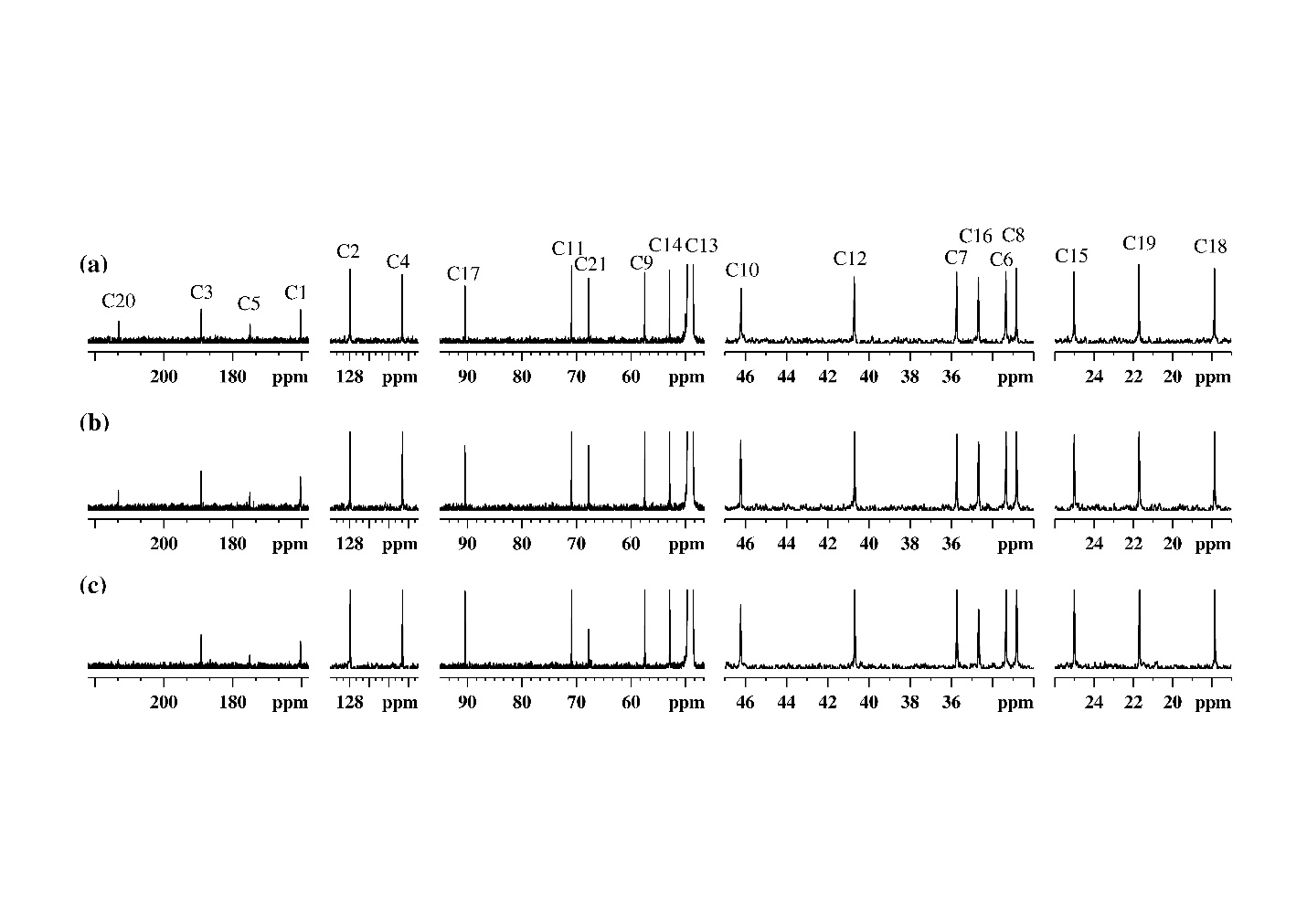


**Figure S3.** Solution ^13^C NMR spectra of Prd/Mg^2+^ mixtures at three different Prd/Mg^2+^ molar ratios: (a) 1:2.5, (b) 1:5.0, (c) 1:7.5. The ^13^C chemical shift assignments are indicated at the top. Some ^13^C resonances revealed mild progressive upfield shifts and others showed progressive downfield shifts as a function of Mg^2+^ concentration. Different regions of the ^13^C spectra are presented on different scales: δ=17-26, 32-47, 50-92, 121-129 and 160-215 ppm.


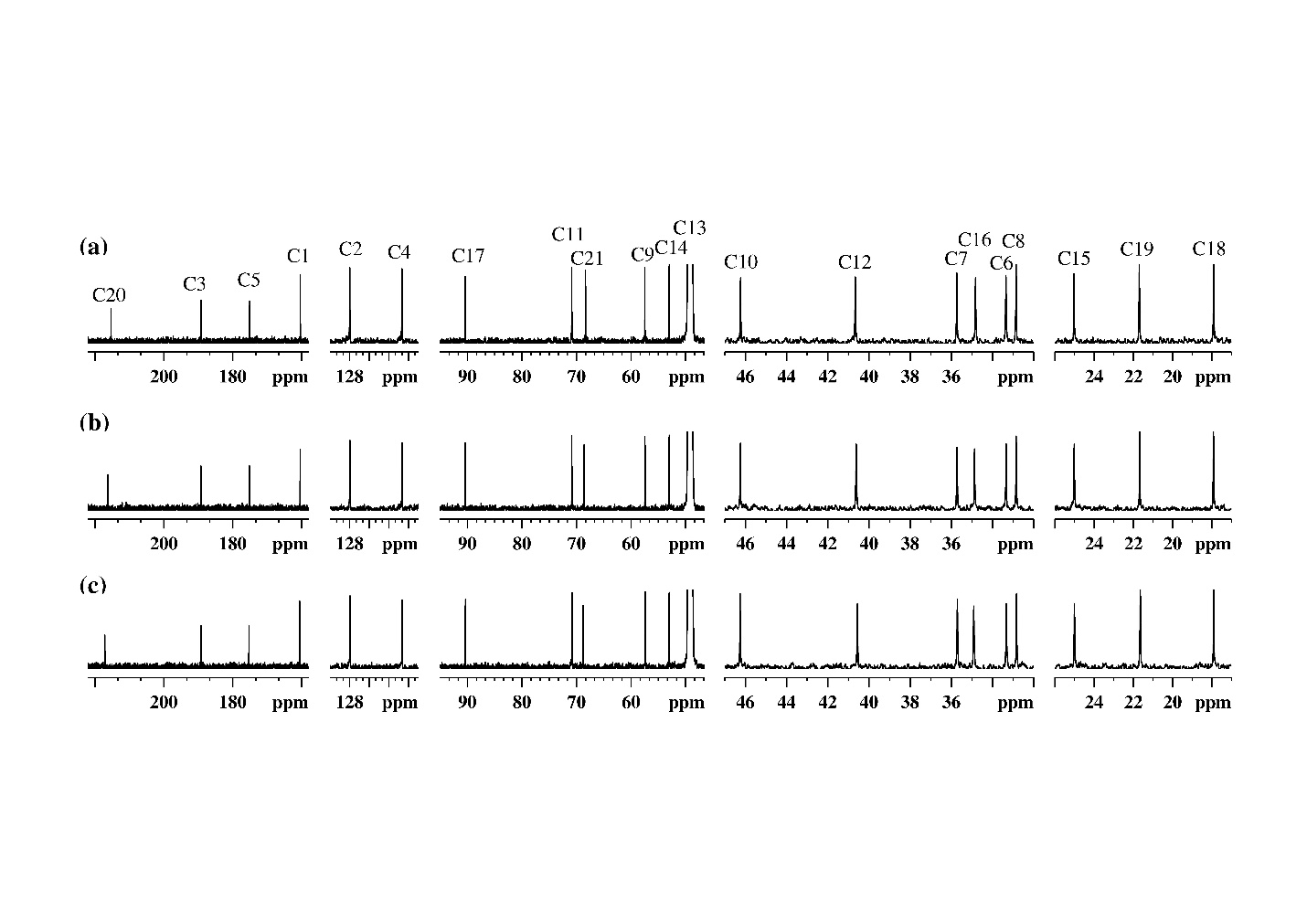


**Figure S4.** Solution ^13^C NMR spectra of Prd/Ca^2+^ mixtures at three different Prd/Ca^2+^ molar ratios: (a) 1:2.5, (b) 1:5.0, (c) 1:7.5. The ^13^C chemical shift assignments are indicated at the top. Some ^13^C resonances revealed mild progressive upfield shifts and others showed progressive downfield shifts as a function of Ca^2+^ concentration. Different regions of the ^13^C spectra are presented on different scales: δ=17-26, 32-47, 50-92, 121-129 and 160-215 ppm.


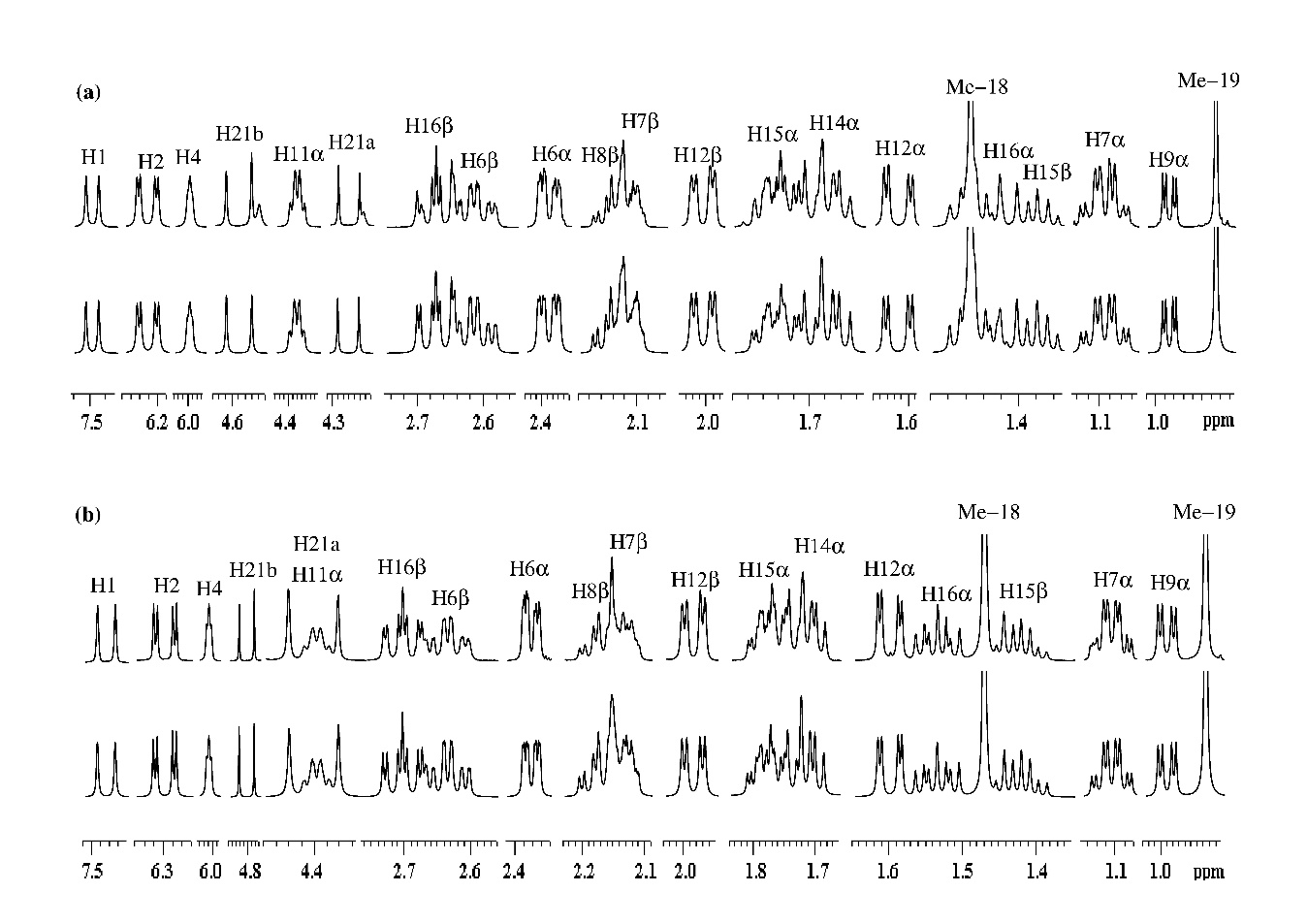


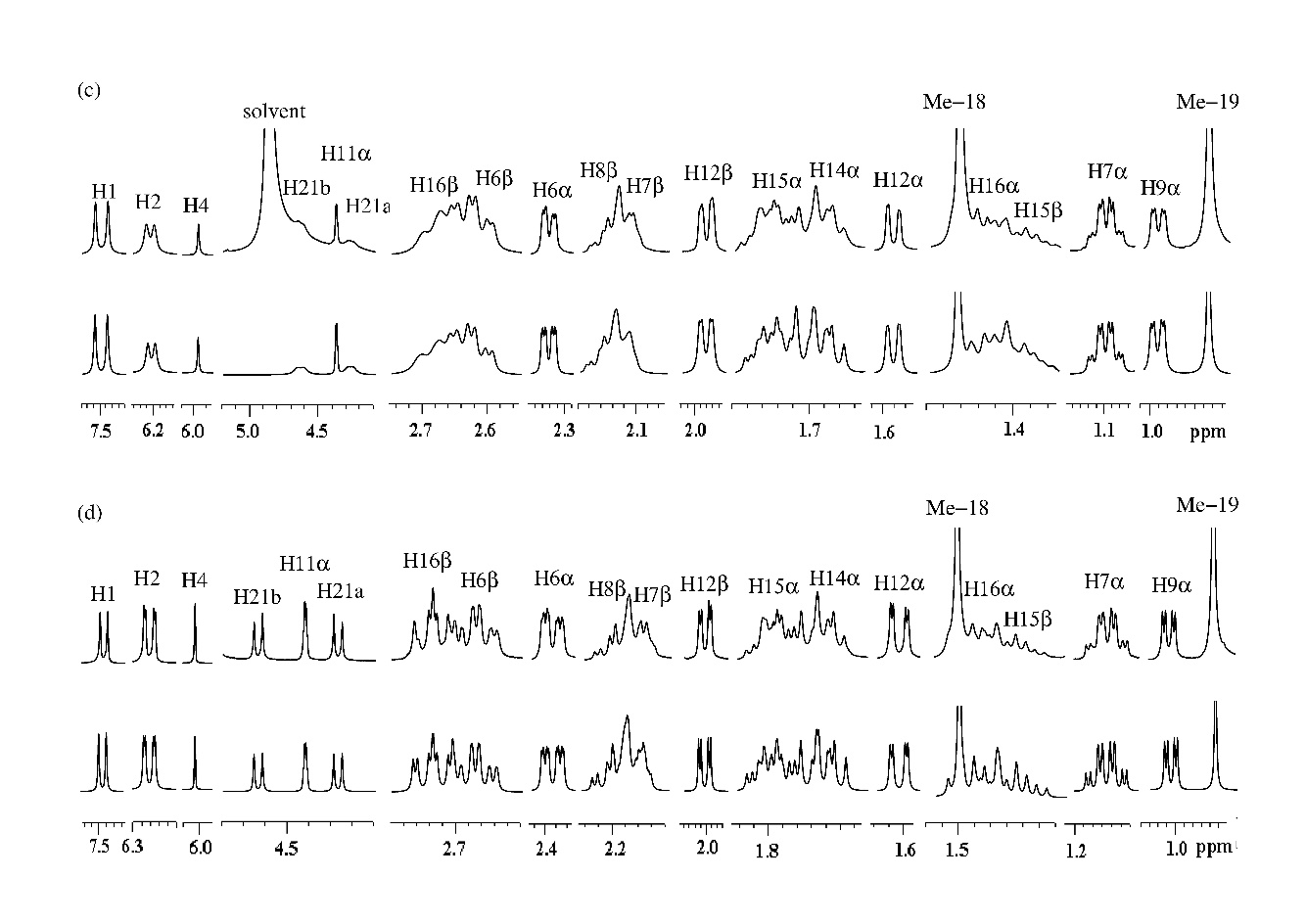


**Figure S5.** Simulation and experimental ^1^H NMR spectra of (a) Prd/Mg^2+^, (b) Prd/Ca^2+^, (c) Prd/Co^2+^, and (d) Prd/Ni^2+^ mixtures. The experimental data was shown on the top trace and the simulation on the bottom. The ^1^H chemical shift assignments are labeled on each of the Prd signal patterns. For better clarity, the ^1^H spectra are displayed in different ranges on different scales.
